# Association Study of Puberty-Related Candidate Genes in Chinese Female Population

**DOI:** 10.1155/2020/1426761

**Published:** 2020-05-22

**Authors:** Gideon Omariba, Junhua Xiao

**Affiliations:** College of Chemistry, Chemical Engineering, And Biotechnology, Donghua University, Shanghai 201620, China

## Abstract

Puberty is a transition period where a child transforms to an adult. Puberty can be affected by various genetic factors and environmental influences. In mammals, the regulation of puberty is enhanced by the hypothalamic-pituitary-gonadal axis (HPG axis). A number of genes such as GnRH, Kiss1, and GPR54 have been reported as key regulators of puberty onset. In this study, we have conducted an association study of puberty-related candidate genes in Chinese female population. Gene variations reported to be related with some traits in a population may not exist in others due to different genetic and ethnic backgrounds, hence the need for this kind of study. The genotyping of SNPs was based on multiplex PCR and the next-generation sequencing (NGS) platform of Illumina. We finally performed association study using PLINK software. Our results confirmed that SNPs rs34787247 in LIN28, rs74795793 and rs9347389 in OCT-1, and rs379202 and rs10491080 in ZEB1 genes showed a significant association with puberty. With the result, it is reasonable to conclude that these genes affect the process of puberty in Shanghai Chinese female population, yet the mechanism remains to be investigated by further study.

## 1. Introduction

Puberty is a period of transition where one turns from childhood to adulthood, hence achieving reproductive capacity [1]. This process takes a period of time and involves a number of events that lead to full activation of reproduction [2]. During this process, secondary sexual characteristics are developed as a result of preeminent secretion of gonadal steroid hormones [3].

Previous studies have shown various gene mutations that disrupt the gonadotropin-releasing hormone, which triggers the onset of puberty [4]. A recent whole-exome sequencing study on 15 families affected with precocious puberty showed mutations on MKRN3 gene in 40 members [5]. Studies have also indicated that MKRN3 can repress puberty onset in mice [6]. It has also been reported by previous genome-wide association studies that single nucleotide polymorphisms (SNPs) near LIN 28B changed the age at menarche [7]. Perry et al. [8] in their GWAs study identified loci which are associated with menarche on women within 3 imprinted genes.

Various advances in high-throughput technologies have indicated that SNPs can influence miRNA's stability and eventually their functional ability [9, 10]. With the advance of high-throughput technology, increasing number of research has revealed that SNPs have profound influence in miRNA function, stability, and targeting [11]. In another genome-wide association analysis of two cohorts, 2 genetic loci were identified near LIN 28B gene. Genome-wide significant associations in two cohort analysis were identified for SNPs in two new genetic loci near LIN28B [12]. Perry et al. [8], in their population meta-analysis on eight cohorts, also identified the same loci near LIN 28B related with age at menarche. Ong et al. [7] also discovered various SNPs associated with puberty near LIN 28 gene. In an earlier candidate gene study, associate FSHB gene has also been associated with age at menarche in earlier candidate gene [12]. A study by Stolk et al. [13] also identified SNPs near five candidate genes that showed significant association with menarche and menopause age.

It could be interesting and of need for future studies to focus on high-throughput sequencing technology, which may be more efficient in functional identifications of genetic variants and their characterization.

## 2. Materials and Methods

### 2.1. Candidate Gene and Variant Selection

In this study, we selected 12 candidate genes based on already published research works. In particular, we selected the 12 genes that have shown significant relationship with puberty as previously reported by other researchers. Thereafter, specific genetic variants single nucleotide polymorphisms (SNPs) were chosen from the known variants based on their linkage disequilibrium (LD).

### 2.2. Participant Recruitment

A random population of 2164 females from Shanghai, China, within the age bracket of 14-25 years was used in this study.

### 2.3. Primer Design

All sequences of the 25 target regions were downloaded from the National Center for Biotechnology Information (NCBI) database (Medha 2010). Specific PCR primers were designed having both target and universal sequences and then set on ideal parameters for PCR reaction. The SNPs and their sequences are shown in Supplementary Table 1.

### 2.4. Two-Round PCR

The PCR reactions used the following program: 94°C for 15 min, 20 cycles of 94°C for 30 s, 60°C for 1 min, and 72°C for 30 s. The PCR products of 2000 samples were then mixed in a 50 ml centrifuge tube after two-round PCR, and then, the tube was sealed by parafilm and mixed overnight. This mixture was purified using the TIANgel Midi Purification Kit (TIANGEN BIOTECH, Beijing, China).

### 2.5. MiSeq v2 Kit

The Illumina MiSeq kit instructions were followed using a 2 × 250 bp paired-end sequencing protocol [14].

### 2.6. Data Analysis

NGS QC Toolkit v2.3 [15] was used for raw reads quality filtering. BWA software was used to demultiplex the filtered reads [16]. SAMtools v1.2 [16] was used to generate pileup file or each sample.

### 2.7. Basic Statistics and Association Study

PLINK software [17] was used in performing the basic statistics and association studies.

## 3. Results

### 3.1. Phenotype Description

**Figure 1:**
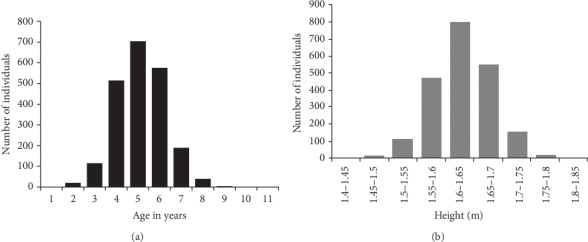
(a, b) Phenotype description of age and height at menarche.

We totally measured the age at menarche and height of 2164 female samples. For the age of menarche, almost 1800 individuals between 12 and 14 years of age attained the menarche phenotype within this period. However, just a few individuals appeared to have the menarche phenotype at the ages of 10, 11, 15, and 16 years. The graphs given in figure 1 a and b represent phenotyping information about the different ages of menarche and heights, respectively. The menarche phenotype is most prevalent between the ages of 12 and 14 years, indicating the highest number of individuals that attained the phenotype. The authors had no significant value for the association of height with puberty. However, when varying heights were compared with menarche, it was noted that individuals between the heights of 1.55 and 1.7 meters seemed to have attained puberty. This is explained by the graphs in Figures 1(a) and 1(b).

### 3.2. Candidate Gene and SNP Selection

In this study, we selected candidate genes depending on the published research work on various potential candidate genes related to puberty. In the recent past, a number of genes related to puberty were identified through association studies and gene expression analysis. Older studies have identified some transcriptional genes of puberty, which we have used as potential puberty genes for our research. The candidate genes we selected are listed in Table 1.

### 3.3. LD Plot

**Figure 2:**
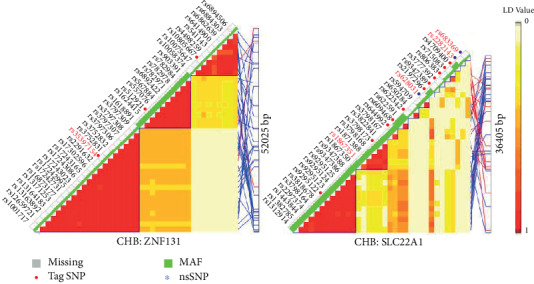
Linkage disequilibrium patterns for SNPs showing close association with high-risk haplotype.

The LD structures of risk SNPs in CHB population from the data of HapMap phase II release 23 is shown in Figure 2. The blocks were constructed with Haploview 4.2 [18].

### 3.4. SNP Selection

The SNP selection summary is shown in Table 2.

### 3.5. Basic Statistics of Genotyping Results

**Figure 3:**
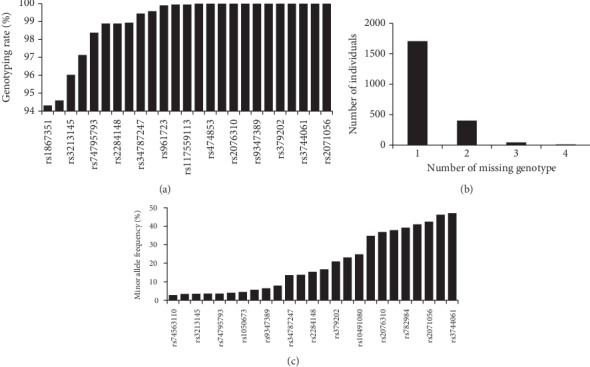
(a) The percentage genotyping rate against the genotyped SNPs. (b) The genotyping result of the number of missing genotype against the number of individuals. (c) The allele frequency results. It indicates that all SNPs attained the minor allele frequency (MAF) of 0.05 and more, which shows that the SNPs are related to the study analysis.

In this study, we genotyped 25 SNPs in 2164 samples totally. The *y* axis represents the percentage of genotyping rate while the *x* axis represents the SNPs genotyped. The result in Figure 3(a) shows that 15 SNPs got 100% genotype and the rest 10 SNPs got a significantly high genotyping rate of above 94%, which indicates that all the SNPs were positively genotyped.

The result as indicated by the graph in Figure 3(b) shows that out of 2164 individuals, 1708 had zero missing genotype, 401 individuals had 1 missing genotype, 44 individuals had 2 missing genotypes, and 10 individuals had 3 missing genotypes. It clearly indicates that nearly all individuals were successfully genotyped with just a few missing genotypes, hence making the result highly efficient for the study.

### 3.6. Association Result

We tested the HWE for all 25 SNPs with PLINK software, and none of them achieved significance (*p* < 0.05), suggesting the population is HWE. The SNPs that are associated with puberty at empirical *p* < 0.05 are represented in Table 2.

Five of the SNPs, rs350115532, rs74795793, rs9347389, rs379202, and rs10491080, with their related genes, show a high significance on puberty, due to their high *p* value. The associated genotypes and alleles are shown in Table 3.

## 4. Discussion

This study focuses on whether the 12 selected genes from already published research works are genetically associated with puberty using a random female population of Shanghai. We evaluated 25 SNPs in the 12 selected genes. Our results show that 5 SNPs have high significant value in relation to puberty compared with the rest of the SNPs. The 5 SNPs have been found in the three genes as shown in Table 3. According to other researchers, the genes have high association with puberty in dynamic populations. Consistent with our findings, a GWAS study has reported SNPs that altered age at menarche near LIN 28B [7]. Similarly, Zambelli et al. [19] identified Oct-1 isoforms within human and mice species. ZEB1 gene has been directly linked to puberty regulation on a transcriptional level by stimulating GnRH gene related with puberty onset [20].

The genes that showed significant association with puberty in this study, LIN28, Oct-1, SLC22A1, and ZEB1, have been reported to be involved in various important biological pathways, such as development, tumorigenicity, immune response, gene expression, and endocrine pathway. It has been discovered that Lin 28 gene has the ability as a heterochronic gene, which plays a crucial role in development [21].

Researchers have also discovered that Lin 28 is associated with embryonic maturation, but its expression has less impact in adults [22]. Oct-1 has been reported to be a coactivator in S phase, a selective recruitment process of G2B promoter which is essential in S phase H2B transcription (Lei et al. 2003). As an essential transcription factor, Oct-1 is widely expressed in various isoforms of Oct-1 in both adults and embryonic tissues of humans and mice [19]. Oct-1 has also been related with regulation of target gene expression and various biological processes in humans and mice [23]. Evidence shows that targeted gene expression can be controlled by extracellular signals which regulate Oct-1 binding properties on DNA like phosphorylation [24], O-GLcNAcylation [25], and ubiquitylation [26]. In the study, ZEB1 repressed GnRH together with other gene encoding transcription factors that commonly promote GnRH expression. ZEB1 encodes to the promoter of the kisspeptin receptor GPR54 through its binding site, hence stimulating the nuclear translocation of OTX2, a transcription factor that promotes GnRH expression [20].

The genes selected in this study have already been reported to have been associated with puberty in different populations. However, in this particular study, we are interested in knowing whether the reported genes are also associated with puberty in the Shanghai female population due to different alleles, populations, or distinct environments.

Out of our 25 selected SNPs, we genotypically identified 5 SNPs (rs350115532, rs74795793, rs9347389, rs379202, and rs10491080) associated with puberty. These SNPs were found in three genes: LIN28, Oct-1, and ZEB1. These findings confirm with other findings reported on these genes having an association with puberty despite different populations. For example, it is recorded that Lin 28 Tg female mice shows a delayed virginal opening and first estrous. There is also a decrease in uterus and ovarian weights. Additionally, the time for the first litter was delayed [27]. Moreover, it has been discovered that Lin 28 can be differentially expressed in both primates and mouse spermatogonia [28]. With all the given evidences, we can conclude that Lin 28/Let-7 system has a profound role in development and puberty onset. However, the metabolic homeostasis of the whole process needs further analysis. Tommiska et al. [29] described that Lin 28-related genes (Lin 28 and Lin 28b) have protein-encoding properties which eventually bind RNA target pairing of zinc finger motifs.

ZEB1 gene has been reported to be encoding the promoter of the kisspeptin receptor GPR54 through its binding site, hence stimulating the nuclear translocation of OTX2, a transcription factor that promotes GnRH expression [20]. Since GnRH expression is known to be the key stimulator of the reproduction process, ZEB1 gene has been directly linked to puberty regulation on a transcriptional level. Oct-1 gene is reported to have the ability to regulate a variety of gene expression which also affects puberty and developmental processes [30].

While in our study we give a substantial contribution to genetic association of the given genes with puberty, we have a limitation whereby we relied on one particular Shanghai sample. We look forward to doing a similar study using various sample populations for more affirmation of the results.

## 5. Conclusion

In conclusion, we establish an association data which agrees with other reported researchers on the association of the three genes, LIN 28, OCT1, and ZEB1, with puberty using a specific Shanghai population. These three genes can be potential candidate genes for future studies on puberty and its mechanisms.

## Figures and Tables

**Table 1:** The 12 candidate genes selected based on previous reports of positive associations with puberty.

Symbol	CHR	Start-end	References
LIN28A	1	26,737,269-26,756,219	
ZNF131	5	43,121,642-43,175,823	
PITX1	5	134,363,424-134,369,964	
COL11A2	6	31,626,992-33,193,009	
RXRB	6	33,161,362-33,168,473	
SLC22A1	6	160,121,789-160,159,201	
HIBADH	7	27,525,440-27,663,001	
ZEB1	10	31,608,101-31,818,742	
MFSD11	17	76,736,565-76,803,805	
USF2	19	35,759,896-35,770,718	
SIX5	19	46,268,043-46,272,497	
E2F1	20	32,263,292-32,274,210	

**Table 2:** The 25 tag SNPs selected showing the genes associated with puberty.

Gene	SNP	CHR	Position (bp)	*p* value		
				Height	Menarche	Menarche (height)
LIN28A	rs35015532	1	26752129	0.4226	0.1778	0.1801
LIN28A	rs34787247	1	26755073	0.06595	0.01336	0.01276
ZNF131	rs80346823	5	43132933	0.2305	0.3686	0.3738
ZNF131	rs782984	5	43172896	0.7196	0.1408	0.1401
PITX1	rs474853	5	134365091	0.5585	0.2348	0.2329
COL11A2	rs1050673	6	33161661	0.1699	0.996	0.9945
RXRB	rs2076310	6	33166034	0.5161	0.2925	0.2952
RXRB	rs117559113	6	33166554	0.8753	0.2214	0.222
SLC22A1	rs1867351	6	160543123	0.8205	0.05056	0.05016
SLC22A1	rs74795793	6	160551101	0.05233	0.007146	0.007602
SLC22A1	rs683369	6	160551204	0.7578	0.6387	0.6369
SLC22A1	rs9347389	6	160575146	0.3908	0.01304	0.01332
HIBADH	rs961723	7	27657973	0.2278	0.4564	0.4492
HIBADH	rs7778454	7	27663276	0.6273	0.2351	0.2335
HIBADH	rs74563110	7	27664814	0.7674	0.1152	0.1158
ZEB1	rs379202	10	31686438	0.5259	0.007294	0.00718
ZEB1	rs10491080	10	31747097	0.7655	0.00693	0.006886
MFSD11	rs3744061	17	74733403	0.7158	0.8719	0.8744
USF2	rs10419959	19	35764705	0.5707	0.2577	0.2601
USF2	rs2284148	19	35765424	0.927	0.478	0.4776
USF2	rs916145	19	35767884	0.4795	0.4065	0.4023
USF2	rs77320927	19	35769378	0.02798	0.0667	0.06406
SIX5	rs2341097	19	46268902	0.3892	0.3635	0.3674
E2F1	rs2071056	20	32265513	0.01422	0.342	0.3306
E2F1	rs3213145	20	32273567	0.984	0.3596	0.3596

**Table 3:** The associated genotypes and alleles.

CHR	SNP	A1 (minor allele)	A2 (major allele)	MAF	
6	rs34787247	A	G	0.13355
6	rs74795793	C	T	0.03642
6	rs9347389	T	C	0.06449
10	rs379202	A	G	0.2097
10	rs10491080	G	A	0.248

## Data Availability

The candidate genes' data supporting this analysis are from previously reported studies which have been cited. The participants' data was obtained from random female population of Shanghai.

## Supplementary Materials

Supplementary Materials Table 1: the 25 SNPs and their respective sequences.
