# Supplementary figures and images for: Association Study of Puberty-Related Candidate Genes in Chinese Female Population
Source: Int J Genomics. 2020 May 22;2020:1426761. doi: 10.1155/2020/1426761 (PMC7285286; doi:10.1155/2020/1426761)

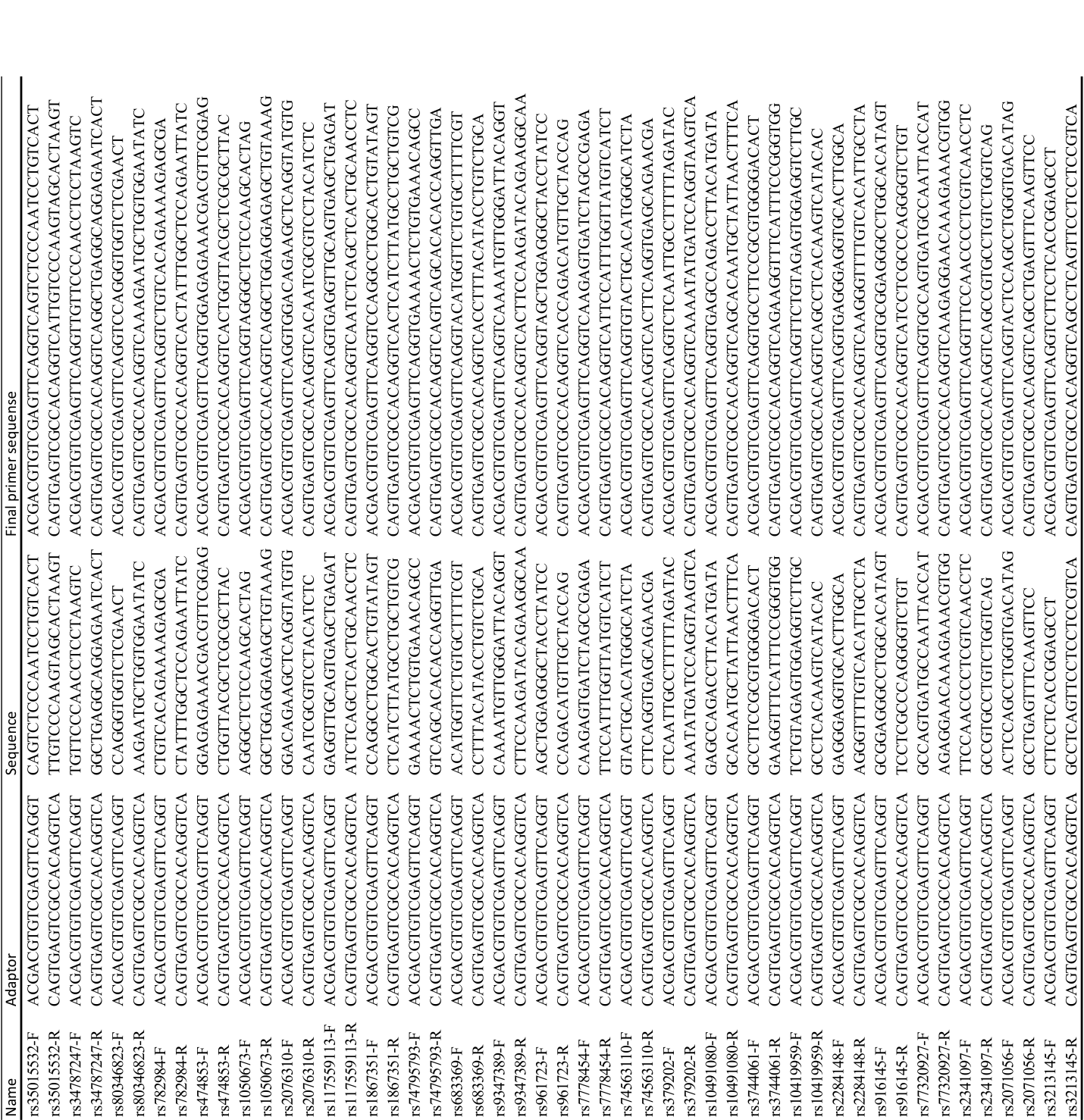


**Supplementary Table 1**. The 25 SNPs and their respective sequences

Supplement: Supplementary Materials — Table 1: the 25 SNPs and their respective sequences. [file 1426761.f1.docx]
